# Softening of the Brain after Coal-gas Poisoning

**Published:** 1870-02

**Authors:** 


### Softening of the Brain after Coal-gas Poisoning.

Th. Simon, of Hamburg, points out the occurrence of cerebral haemorrhage and softening, after inhalation of the mixed gases arising from the combustion of coal. Between the date of poisoning and the appearance of cerebral symptoms a considerable interval may apparently elapse. In one case, cited from Oppolzer, the patient spat blood for one day after the accident, then appeared well and able to work for eight days, and finally returned to the hospital with headache and great disturbance of speech, from which he did not recover for several months. In Case II., the patient suffered from giddiness, lasting three or four days, and headache, which, though continuous, did not prevent him from resuming work when the giddiness had passed off. The pain became localized in the left parietal region. In one month he was struck with apolexy, and died on the same day. The middle of the left cerebral hemisphere, the corpus striatum, and thalamus opticus, were found softened to an atheromatus condition. In Case III., the poisoning was voluntary; the man had shown previous symptoms of aberration. He was recovering his speech and control over his limbs when, after the lapse of several days, somnolence and paralysis suddenly set in, and increased until his death. A large portion of the left hemisphere was found softened and slightly yellow. In Case IV., a woman was poisoned by gas from an iron stove, and recovered with difficulty. She was tolerably well for ten days, and then, after symptoms of mental derangement lasting eight days, was brought to hospital, with general paralysis and spasms. Death on the third day. Softening of both corpora striata. In Case II., III., IV., atheroma of the cerebral arteries was not observed.

Ackerman has demonstrated by experiment that poisoning by carbonic oxide and illuminating gas is accompanied with intence hyperaemia of the brain. Letheby found almost constantly, in birds, a haemorrhage into the brain. The author has examined, *post mortem*, sixty cases of coal-gas poisoning; in forty-eight of these the brain and its membranes were hyperaemic; in foouteen, excessively so. In rare instances, great anaemia is found.

In explaining the processes that have been observed in the cases quoted, it may be assumed that the first stage of red softening commences during the actual asphyxia, or the so-called "reactive inflamation" may occur subsequently in the parts surrounding a small focus of softening; or the headache may be looked upon as a symptom of disturbed nutrition, caused by the carbonic oxide, and leading to softening; or the process may depend on fatty degeneration in the arteries of the brain, such as is known to accompany carbonic oxide poisoning in many other organs.

